# The complex health seeking pathway of a human African trypanosomiasis patient in Côte d’Ivoire underlines the need of setting up passive surveillance systems

**DOI:** 10.1371/journal.pntd.0008588

**Published:** 2020-09-14

**Authors:** Minayégninrin Koné, Emmanuel Kouassi N’Gouan, Dramane Kaba, Mathurin Koffi, Lingué Kouakou, Louis N’Dri, Cyrille Mambo Kouamé, Valentin Kouassi Nanan, Gossé Apollinaire Tapé, Bamoro Coulibaly, Fabrice Courtin, Bernardin Ahouty, Vincent Djohan, Bruno Bucheton, Philippe Solano, Philippe Büscher, Veerle Lejon, Vincent Jamonneau

**Affiliations:** 1 Unité de Recherche « Trypanosomoses », Institut Pierre Richet, Bouaké, Côte d’Ivoire; 2 Laboratoire de Biodiversité et Gestion des Ecosystèmes Tropicaux, Unité de Recherche en Génétique et Epidémiologie Moléculaire, Université Jean Lorougnon Guédé, UFR Environnement, Daloa, Côte d’Ivoire; 3 Projet de Recherches Cliniques sur la Trypanosomiase (PRCT), Daloa, Côte d’Ivoire; 4 Programme National d’Élimination de la Trypanosomose Humaine Africaine, Abidjan, Côte d’Ivoire; 5 Direction Départementale de la Marahoué, District sanitaire de Sinfra, Ministère de la Santé et de l’Hygiène Publique, Abidjan, Côte d’Ivoire; 6 Direction départementale de la santé de la Marahoué, Centre de Santé Urbain de Bonon, Ministère de la Santé et de l’Hygiène Publique, Abidjan Côte d’Ivoire; 7 Unité Mixte de Recherche IRD-CIRAD 177, INTERTRYP, Institut de Recherche pour le Développement (IRD) Université de Montpellier, Montpellier, France; 8 Department of Biomedical Sciences, Institute of Tropical Medicine, Antwerp, Belgium; Makerere University, UGANDA

## Abstract

**Background:**

Significant efforts to control human African trypanosomiasis (HAT) over the two past decades have resulted in drastic decrease of its prevalence in Côte d’Ivoire. In this context, passive surveillance, integrated in the national health system and based on clinical suspicion, was reinforced. We describe here the health-seeking pathway of a girl who was the first HAT patient diagnosed through this strategy in August 2017.

**Methods:**

After definitive diagnosis of this patient, epidemiological investigations were carried out into the clinical evolution and the health and therapeutic itinerary of the patient before diagnosis.

**Results:**

At the time of diagnosis, the patient was positive in both serological and molecular tests and trypanosomes were detected in blood and cerebrospinal fluid. She suffered from important neurological disorders. The first disease symptoms had appeared three years earlier, and the patient had visited several public and private peripheral health care centres and hospitals in different cities. The failure to diagnose HAT for such a long time caused significant health deterioration and was an important financial burden for the family.

**Conclusion:**

This description illustrates the complexity of detecting the last HAT cases due to complex diagnosis and the progressive disinterest and unawareness by both health professionals and the population. It confirms the need of implementing passive surveillance in combination with continued sensitization and health staff training.

## Author summary

Human African trypanosomiasis (HAT) or sleeping sickness is a parasitic disease caused by *Trypanosoma brucei* that is transmitted by tsetse flies. In 2012, HAT was included in the World Health Organization roadmap for the control of neglected tropical diseases with the objective of elimination as a public health problem by 2020. In Côte d’Ivoire, HAT prevalence has dropped sharply the last decade. A passive HAT surveillance was therefore integrated in the national health system, which allowed to detect a first patient in 2017. This article describes the complex health seeking pathway and suffering before diagnosis of this patient, an 11 years old girl, and illustrates the challenge when health agents and population no longer consider HAT as a threat in an elimination context. Our results show the need to install a solid surveillance system, in combination with continued sensitization and repeated health staff training.

## Introduction

Human African trypanosomiasis (HAT) or sleeping sickness is a neglected tropical disease caused by the protozoan parasites *Trypanosoma brucei gambiense* (*Tbg*) and *Tb rhodesiense* (*Tbr*) which are transmitted by tsetse fly (*Glossina spp*) bites. The disease occurs in Sub-Saharan Africa, within the limits of its vector distribution [1]. Thanks to the intensive control efforts deployed since the early 2000s, the number of HAT cases has evolved from 26550 in the year 2000 to 977 in 2018. However, still 57 million people are at risk for infection. As defined in its roadmap, WHO has targeted HAT elimination as a public health problem by the year 2020, and interruption of transmission by 2030 [2].

Côte d’Ivoire is at present the second HAT affected country in West-Africa, with endemic foci in Central-Western forest areas [3,4]. Rural populations living from handwork in cash (cocoa, coffee) and subsistence (banana, rice) agriculture are the most affected. For a long time, the HAT control strategy in Côte d’Ivoire has been based on active case detection by mass screening using the Card Agglutination Test for Trypanosomiasis (CATT) [5], followed by parasitological examination of seropositive individuals [6]. This strategy, implemented by mobile teams who travel from village to village, has allowed to drastically reduce the number of detected HAT cases, with less than 10 yearly cases since 2009 [2,4,7].

However, in a low prevalence context, this strategy is not cost effective. The population does not consider HAT as a threat anymore and refuses to participate to mass screening activities [8,9]. The fact that in 2010 and 2011 not a single case of HAT was found during active screening, while 12 HAT patients were diagnosed in only one national treatment centre [7], demonstrates well the need of implementing a passive HAT surveillance based on clinical suspicion integrated in the national health system [10,11,12]. This need is also confirmed by the itinerary of a HAT patient that we describe here, illustrating the progressive disinterest and unawareness by both health professionals and the population as HAT is being eliminated.

## Materials and methods

### Study area

**Fig 1:**
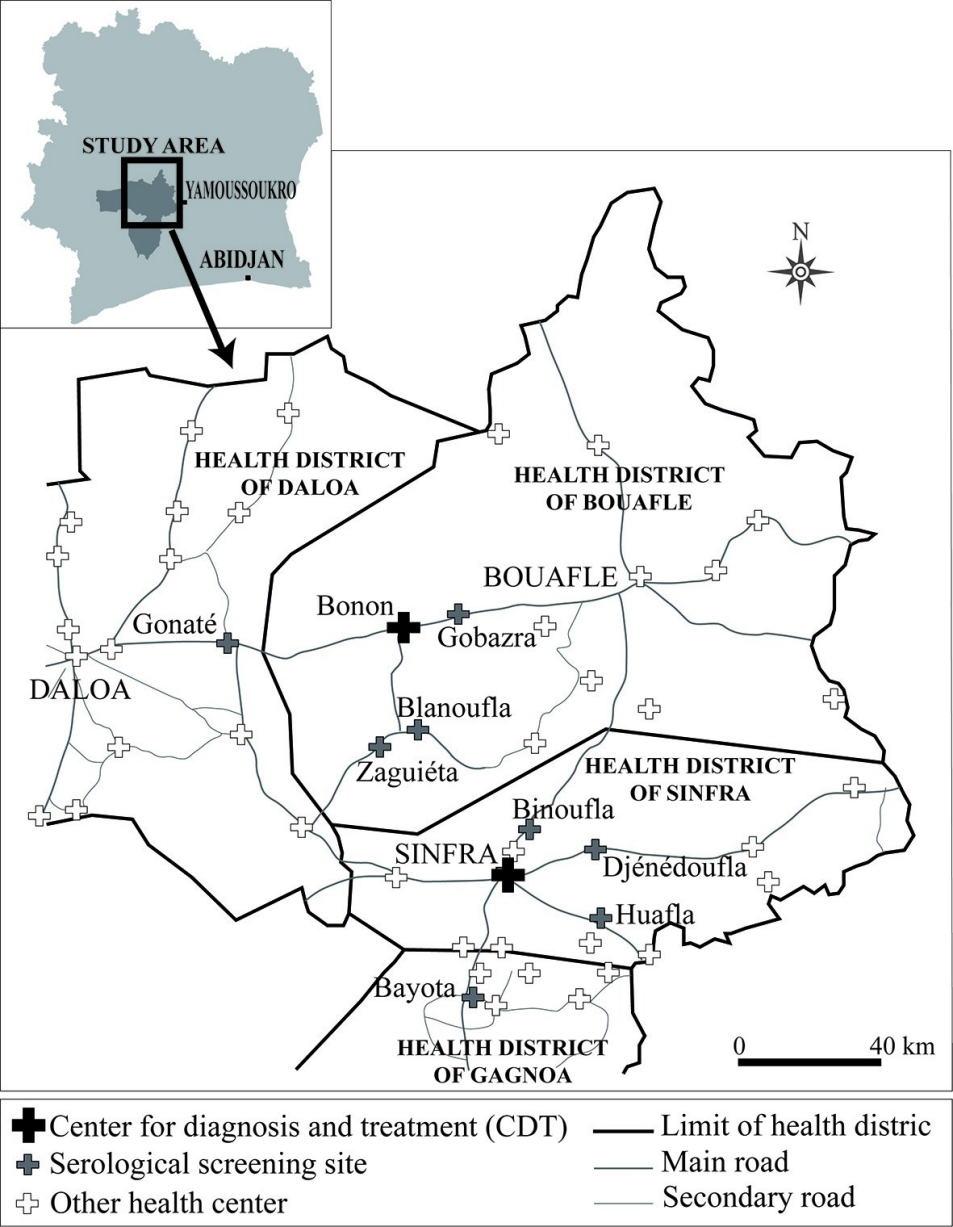
Map showing the localisation of the passive surveillance sites implemented in the Bonon and Sinfra HAT endemic foci in Côte d’Ivoire in 2017.

The national health system in Côte d’Ivoire is organized as a classical three level pyramid, with health centres that serve as an entry point at the basis, general hospitals at the secondary level and university hospitals and specialized institutes at the tertiary level. These public health structures are complemented by private clinics and hospitals as well as a network of traditional medicine officially recognized by the Ministry of Health[13]. Until 2017, passive HAT surveillance in Côte d’Ivoire relied on two HAT treatment centres: the Projet de Recherches Cliniques sur la Trypanosomiase in Daloa (PRCT) and the Base de Santé Rurale in Bouaflé (closed in 2006) (Fig 1). In August 2017, passive surveillance was extended to the two last endemic foci of the country, Bonon and Sinfra, within the framework of a diagnostic clinical trial (DiTECT-HAT) evaluating the performance of diagnostic tools and algorithm. Ten health structures were selected at the primary level (Fig 1). Two structures, the Sinfra Hôpital Général (HG) and the Bonon Centre de Santé Urbain (CSU), were mandated to perform clinical and serological screening as well as parasitological confirmation and treatment (centres for diagnosis and treatment). The eight other were health centres performed clinical and serological screening only, and referred seropositive suspects to one of the two centres for confirmation diagnosis.

### Diagnostic methods for passive surveillance in Bonon and Sinfra

The diagnostic procedure was initiated by clinical suspicion for HAT. The following symptoms were considered: long-lasting fever resistant to anti-malaria treatment, long-lasting headaches (>14 days), swollen cervical lymph nodes, weight loss, weakness, intense pruritus, amenorrhea, abortion, sterility, coma, psychiatric problems (aggressiveness, apathy, mental confusion, hilarity), sleep disruption (nocturnal insomnia and excessive daytime sleepiness), motor problems (convulsions, abnormal movements, tremor, walking difficulties) and speech difficulties. An individual was a clinical suspect if at least one of these symptoms was present, which initiated serologically testing for presence of specific antibodies. Within the DiTECT-HAT diagnostic trial, three rapid diagnostic tests (RDT) were carried out simultaneously: SD Bioline HAT 1.0 (Standard Diagnostics, Korea, [14]), HAT Sero-*K*-Set (Coris Bioconcept, Belgium, [15]) and rHAT Sero-Strip (Coris Bioconcept, Belgium, [16]). Clinical suspects positive in at least one RDT, underwent parasitological examination by the mini-anion exchange centrifugation technique (mAECT) performed on whole blood [17, 18] and for those who had swollen cervical lymph nodes, a microscopic examination (x400 magnification) of lymph node fluid. Dried blood spots were also prepared for further testing in immunotrypanolysis [19], enzyme-linked immunosorbent assay (ELISA/*Tbg* [20,21]), loop-mediated isothermal amplification (LAMP [22]), and real-time PCR targeting the 18S rRNA and the *Trypanosoma brucei gambiense*-specific glycoprotein (TgsGP) genes specific respectively for *Trypanozoon* [23] and *Tbg* [24]. Patients in whom trypanosomes were detected were treated according to the national procedures and WHO recommendations [25].

### Epidemiological investigations

For each detected HAT case, a spatial and geo-referenced investigation of the travel history and living places was carried out to identify populations that share the same environment and are at increased risk for HAT [4, 26]. An epidemiological investigation was carried out to describe in detail the clinical evolution and the health and therapeutic itinerary of the patient (discussions with the family, discussions with health agents that examined the patient, consultation of all possible documentation such as the health book, the results of different analyses, hospitalisation certificates, prescriptions etc.)

### Ethics

Before commencement, the DiTECT-HAT diagnostic trial (Diagnostic Tools for Human African Trypanosomiasis Elimination and Clinical Trials, work package 2, passive case detection) received ethical clearance (i) from the Comité Consultatif de Déontologie et d'Ethique of the French National Institute for research on sustainable development (IRD), (ii) from the Institutional Review Board of the Institute of Tropical Medicine Antwerp Belgium (reference 1133/16), (iii) from the Ethics Committee of the University of Antwerp (Belgian registration number B300201730927) and (iv) from the Comité National d’Ethique de la Recherche, Ministry of Public Health and Hygiene in Côte d’Ivoire (reference 076//MSHP/CNER-kp). The DiTECT-HAT trial has been registered at ClinicalTrials.gov, identifier NCT03356665. Before being included in DiTECT-HAT, the study was explained to the patient and her mother and written ascent and informed consent was obtained. A written ascent and informed consent was also obtained to study and describe the pathway of the HAT case in the frame of the HAT elimination project in Côte d’Ivoire for which ethical clearance was received from the Comité National d’Ethique de la Recherche, Ministry of Public Health and Hygiene in Côte d’Ivoire (reference 030-18/MSHP/CNER-kp).

## Results

The patient was the first one diagnosed by the passive surveillance activities implemented in the two endemic foci in Côte d’Ivoire. The patient was an 11 years old girl detected in September 2017 in the Bayota CSU, a serological screening site in the Sinfra focus.

### Diagnosis at inclusion

The girl presented with long-lasting fever that did not respond to malaria treatment, serious weight loss, weakness, sleep disruption, motor problems (tremor and unable to walk), anorexia and speaking difficulties. All three RDT performed in Bayota were positive (S1 Fig), and the girl was referred to the Sinfra HG for parasitological examination. In the absence of swollen cervical lymph nodes, parasitological confirmation was carried out by examination of blood in the mAECT, with only one trypanosome observed (S2 Fig). The patient was diagnosed with stage 2 HAT, having 160 white blood cells/μl and trypanosomes in the cerebrospinal fluid and was successfully treated using Nifurtimox Eflornithine Combination Therapy in the Bonon CSU since the Sinfra HG was, at that time, not yet functional for HAT treatment. Her dried blood spots were positive in immune trypanolysis, ELISA and LAMP but negative in real time PCR.

### Geographical itinerary

**Fig 2:**
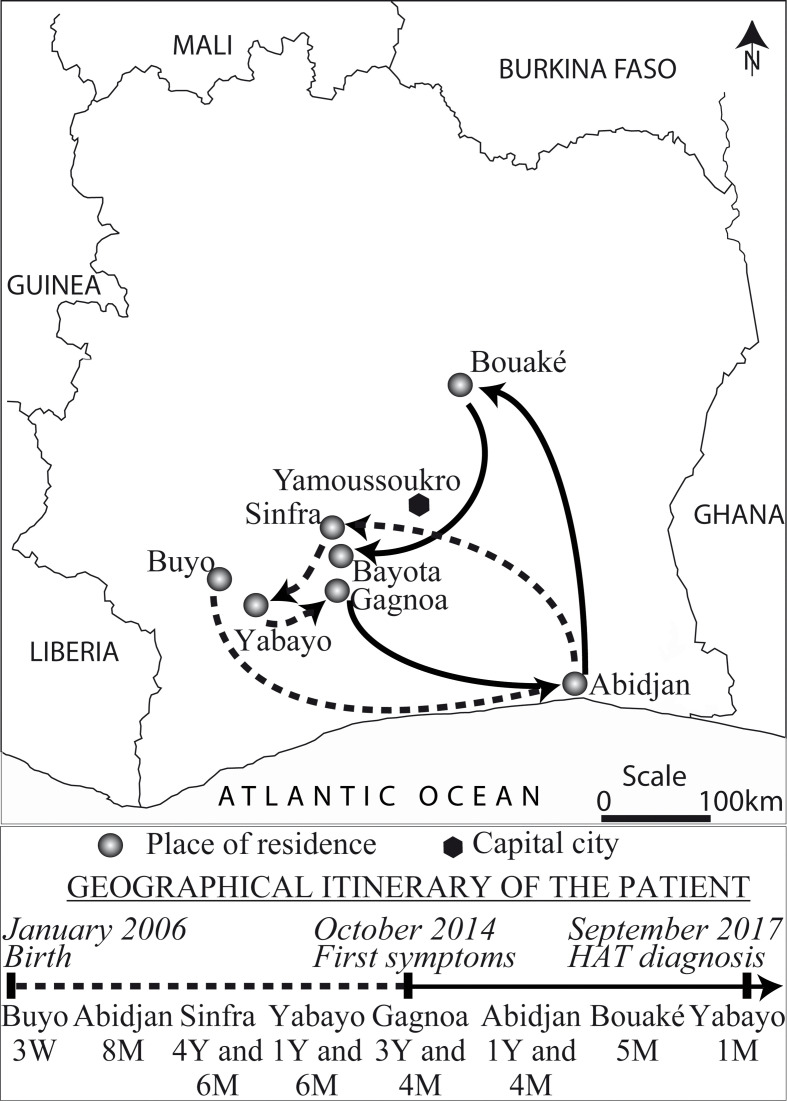
Geographical and health itinerary of the patient between birth and HAT diagnosis. W = week(s), M = month(s), Y = year(s).

The girl was born in January 2006 in Buyo, situated in South-West Cote d’Ivoire in the department of Soubré, in the Nawa region (Fig 2). Three weeks after her birth, her mother took her to Abidjan, from which 8 months later, they moved to Sinfra where they stayed for 5 years. In March 2011, she moved with the girl to Yabayo, a city in Central-West Côte d’Ivoire to do business. In September 2012, they went to Gagnoa, where the girl went to school and was under the supervision of her grandmother. The mother installed herself to work in Bouaké, a city in the centre of the country. In December 2015, the girl dropped out of school due to the deterioration of her health (see below). In January 2016, the mother left her job in Bouaké to seek health care for the girl in Abidjan where they stayed with the mother's family. Running out of financial means, they went to Bouaké in April 2017, for the mother to resume her job, while looking for a cure for the girl. After numerous examinations and despite a strong HAT suspicion established in Bouaké (details provided below), the mother, decided in August 2017 to take her girl to Bayota (20 km south of Sinfra) where her husband resided. It was at the Bayota CSU and then at the Sinfra HG that she was re-screened in September 2017 after which the diagnosis could be confirmed.

### Clinical evolution and health seeking itinerary

The first clinical signs of HAT date back to October 2014 (Fig 2) with fever and headaches that were treated with traditional self-medication. In November 2014, the same symptoms persisted accompanied by general asthenia. The patient then consulted the nearest primary health care centre, the maternal and child protection centre (PMI) in Gagnoa where malaria was diagnosed and treated. Between December 2014 and July 2015, the PMI continued to diagnose and treat for malaria with monthly intervals, based on the same symptoms, which from June 2015 were accompanied by convulsions. This was the reason for visiting a traditional healer in August. Although from September to October 2015 all symptoms subsided, they reappeared in November 2015, together with hallucinations and behavioural problems (bulimia, hilarity, strong restlessness, abnormal movements). The patient was then hospitalized for 12 days in a private clinic in Gagnoa, but no improvement was observed. In December 2015, the patient's family called upon another traditional healer and a spiritual healer who attributed the troubles to a spirit called "DRAGON". Despite prayers and new treatments, symptoms worsened with appearance of limb paralysis.

In January 2016, the patient and her mother went to Abidjan and first consulted private structures. A strong anemia led to a sickle cell test, which turned out negative. Following the onset of severe fever and diarrhoea, typhoid fever was diagnosed and treated, and anti-anemic drugs were given. In April 2016, the existing symptoms persisted, with the addition of abnormal movements, trembling of the limbs and sleep disturbances (daytime hypersomnolence and night time insomnia). The patient was then admitted to a private clinic. An analysis report and the patients’ health record mention a HAT test, which consisted of a fresh blood examination, which was negative. Anti-anemics and antipyretics were again prescribed. In October 2016, the patient consulted a referral hospital of the tertiary level in Abidjan with, in addition to the aforementioned symptoms, eating difficulties. She was admitted to the neurology department and then to the trauma department. Leg massages were prescribed, as well as medicines for fever and poor appetite. Without improvement, in January 2017, the patient consulted another private clinic in Abidjan where she was diagnosed with malaria and treated.

Back in Bouaké in April 2017, the persistent symptoms associated with severe anemia made the patient to consult an urban health centre where she was once again prescribed antimalarials, antibiotics against typhoid fever and anti-anemics. In July 2017, with the worsening of motor disorders, a generalized paralysis (hypertonia of the limbs), anorexia, almost continuous hyper-somnolence and loss of consciousness, she was referred to the Bouaké CHU where she was admitted to the service of neurology. It is in this department that a precise clinical description was made, described elsewhere [27]. The first real clinical suspicion of HAT was made by a neurologist who knew the symptoms of the disease and who guided the patient to the Institut Pierre Richet, an institution of the National Institute of Public Health (INSP) specialized in research on vector-borne diseases including HAT. Although the RDT and the CATT were positive for presence of specific antibodies, the patient could not be confirmed by parasitological examination in the mAECT. Since a lumbar puncture was performed the day before at Bouaké CHU to perform a direct microscopic observation of cerebrospinal fluid (CSF) which turned out to be negative for trypanosomes, the lumbar puncture was not repeated at this occasion. The patient was invited to come back the next week to repeat the parasitological investigations. Unfortunately, the mother, who lost hope of recovery and trust in conventional medicine, decided to return to Bayota. It was thanks to the strong awareness-raising of the HAT team of IPR who stayed in contact by phone with the mother and to the fact that Bayota was included in the HAT passive surveillance system that she finally agreed to go to the CSU of Bayota in September 2017. The confirmation of the serological suspicion of HAT convinced the mother to go to the Sinfra HG where her daughter was finally parasitologically confirmed.

### Financial aspects

We made an estimation of the total expenditures made by the patient's family between the time of her first symptoms (October 2014) and her treatment (September 2017). We took into account the costs of consultations, the cost of laboratory analyses and the cost of drugs that were prescribed and purchased. We also estimated the mother's loss of income due to the interruption of her professional activities (Table 1). We did not include the transport costs and the costs of living outside the main residence. The total amount spent was 3 718 500 CFA francs or 5 669 Euros. For comparison, the gross domestic product per capita of Côte d’Ivoire was 1 383 Euros in 2017 (https://data.worldbank.org/indicator/NY.GDP.PCAP.CD?locations=CI).

**Table 1:** Details of the expenses for case management of the patient between October 2014 and September 2017.

ITEM	Amount in FCFA*	Amount in EURO
Consultation costs	293 500	447,44
Drug costs	965 000	1 471,13
Costs of medical laboratory analyses	540 000	823,23
24 months’ salary loss of the mother**	1 920 000	2 927,02
**TOTAL**	**3 718 500**	**5 668,84**

* FCFA = currency in Côte d’Ivoire (1 euro = 655,957 FCFA)

** The mother had to stop working for 24 months to take care of her daughter

## Discussion

We examined the clinical, diagnostic, geographical, and financial aspects of a HAT case detected in Côte d’Ivoire. Only one single patient is described, which is clearly a limitation. Although interesting for its peculiarities, it is not possible to extrapolate some of the conclusions to the generality. However, the patient overall pathway clearly demonstrates the difficulties that can be encountered to arrive at the correct diagnosis within a HAT elimination context.

Clinically, symptoms and signs for sleeping sickness are not specific [25]. The first symptoms observed (fever, headache, asthenia, anemia and digestive disorders) were attributed several times to malaria or typhoid fever, confirmed or not by laboratory analyses. The treatments prescribed during three years were in line with these diagnoses and mainly composed of antipyretics, antibiotics, antimalarials and anti-anemics, even after the onset of neurological disorders, since no alternative diagnosis could be made. Suspicion of HAT was raised for the first time in a private clinic in Abidjan, on the basis of sleep disruption, but as the diagnosis could not be confirmed at that time, suspicion was not maintained. It was only at a very advanced disease stage that an experienced neurologist strongly suspected HAT [27] and referred the patient to a specialized structure. The evolution of the patient's clinical signs with three years between the appearance of the first symptoms and an almost comatose state in which she was diagnosed, perfectly illustrates the chronic nature of HAT due to *Tbg* with a slow evolution and a progressive appearance of neurological disorders [28,29].

Also laboratory diagnosis [6], which is needed to initiate treatment for HAT, proved not to be easy. The first parasitological examination, the wet blood film examination, was negative but this technique has a poor sensitivity [30, 31]. The final confirmation was also retarded by 2 months due to a negative mAECT result, despite the fact that mAECT is the most sensitive parasitological method [32,33,34]. During the screening of the patient in September 2017, the three RDT were positive, but only one trypanosome could be detected in blood by the mAECT. This can be explained by the low parasite numbers present in the blood, which is characteristic for *Tbg* HAT, and shows the interest of repeating the parasitological examination [30]. The tests carried out on dried blood spots by the reference lab were positive with exception of the 18S and TgsGP PCR, which can be explained by the limited quantity of DNA on the filter paper [33,35]. In contrast to the blood results, many trypanosomes were observed upon cerebrospinal fluid examination, a phenomenon that is not uncommon in advanced stage HAT [33].

The travel history of the patient contributed to delay diagnosis as well. She spent her early childhood in Sinfra, a well-known HAT focus [3,36,37,38,39], where she was probably infected. Although Yabayo and Gagnoa are historic HAT foci, infection there seems less probable due to her short stay in Yabayo and an exclusively urban life as schoolgirl in Gagnoa, with little probability of exposure to tsetse flies. Unfortunately, the patient did not attend any active HAT screening session before leaving Sinfra, and her chances of being diagnosed, despite the onset of symptoms, were limited in Gagnoa where since 1994 no case of HAT had been detected [37].

From a service provider delay perspective, the patient consulted during 3 years at least twenty health centres in the public and private sector. She used the three levels of the national public health system pyramid and met with more than a hundred doctors, nurses, laboratory technicians and community health workers. This illustrates another difficulty, which comes from the fact that HAT is a neglected tropical disease, whose control, in the absence of vaccines or prophylactics, is based on case management often carried out by specialized teams on the edges of the national health system [40]. In Côte d'Ivoire, for example, control has long relied on two research institutions: Institut Pierre Richet in Bouaké and PRCT. Since 2006, PRCT has been the only centre for passive screening and treatment in the country [3,4,7]. This system offers only very limited access to specialized care, especially in rural areas where HAT mainly occurs [41]. It also limits the number of health workers capable of suspecting the disease, which in addition, is going down in a context of sharply decreasing prevalence. Today, HAT is very succinctly dealt with in the training of health personnel, whatever the level of training is. In addition, a large majority of health workers in Côte d'Ivoire considers HAT as a disease of the past century.

Unfortunately, this is also the case for at-risk communities who no longer consider the disease as a threat, and no longer participate in active screening sessions -hence their ineffectiveness [8] and who gradually lose the reflex of thinking about HAT. Neurological disorders are often attributed to a mystical cause and patients are referred to traditional and spiritual healers. Implication of traditional healers is probably common in diagnostic pathways of patients with rare diseases in an elimination context, but not as well documented for *Tbg* HAT [42], as for *rhodesiense* HAT [43], [44]. These steps contributed to patient delay in seeking the diagnosis. Actions should be taken to raise awareness of the traditional healers so that they can refer clinical suspects to health centres where HAT passive surveillance is implemented. Such an approach would compensate for the often-low presentation rate in health centres in remote areas that characterize the HAT foci [12].

The combination of all difficulties highlighted above, resulted in a patients and service provider delay which was much higher than the median time previously reported from Democratic Republic of Congo [45], which could possibly be attributed to the relatively high HAT prevalence at time of the study in DR Congo, compared to the elimination context in Côte d’Ivoire some years later. The delays led to late treatment of the patient who suffered a lot and still has neurological sequels when writing the manuscript. The present experience also illustrates the financial burden of HAT on families in particular when the suspicion index is low and diagnostic capacities are absent. The costs incurred, including the mothers salary loss for two years, were also much higher than the 44 US$ previously described for DR Congo [45], and led the mother to seek for financial help from her family.

In a context of low HAT prevalence, the implementation of an effective passive surveillance integrated into the national health system [12] could help to overcome some of these difficulties. First, it reminds the health workers of the existence of HAT and gives them the means to screen for it using a simple protocol based on clinical and serological suspicion using RDT [46]. It makes care more accessible to all populations at risk, even the most remote, and targets clinical suspects even if they came to consult for other reasons. Finally, geographically localisation of confirmed patients, enables identification of the areas of transmission and improved orientation of the control efforts. Based on the clinical symptoms, the patient might have been diagnosed earlier, if a passive surveillance system had been in place. In Côte d’Ivoire, the national programme for elimination of HAT has decided to reinforce the passive surveillance system from 2018 on, by extending it to all hypo-endemic foci, which were defined as having one case detected between 2000 and 2015, and implemented it in 13 additional health centres. In addition to primary health care centres, passive surveillance has also been integrated in five tertiary level health centres including in the main psychiatric and neurological services (Abidjan, Bouaké and Bingerville). These centres receive patients from all over the country, as was the case for the patient who consulted two of them, and contribute to national surveillance. This is important as the population at risk for HAT in West-Africa, is often characterized by significant mobility [7,36]. This population has in general very little chance to be tested for HAT when it leaves endemic areas.

Passive surveillance however has some limitations. It is based on clinical suspicion, and risks to detect patients mainly when they present with neurological symptoms and therefore are already in an advanced disease stage [7]. This is an important constraint, not only for the patient, but also for the community since the subject constitutes a reservoir of parasites and a source of contamination for a long time before being detected and treated. To detect additional cases as early as possible, passive surveillance should therefore be accompanied by adapted reactive active screening strategies, such as reactive door to door screening, [4], spatial tracking [26], follow-up of seropositive subjects [47,48,49,50] or identification of villages at risk [51]. It is also essential to inform the health personnel that, even if HAT prevalence has decreased, the disease still exists and carries a risk to re-emerge [52]. Health personnel needs to be trained continuously and must receive the means to diagnose the disease. Equally important, public awareness on HAT should be raised, at the local level in the areas at risk but also at national level, including information on the possibility to be screened in primary health centres. Difficulties in passive case detection of HAT in the context of a significant drop in prevalence have also been observed in DR Congo [53]. In this country, the most affected by HAT [2], a national HAT commemoration day has been created [54], a sensitization initiative that could be extended to all endemic countries.

In conclusion, the detailed analysis of the clinical, diagnostic, geographical, and financial aspects of this HAT case, whose diagnosis was facilitated by a clinical trial, underlines both the complexity and the importance of detecting the last HAT cases and of reaching HAT elimination in Côte d’Ivoire where tsetse flies are found throughout the territory [55]. Reinforcement of passive surveillance at national scale, integrated in the existing health system could help to overcome some difficulties in combination with continued sensitization and health staff training.

## Supporting information

S1 Fig Photo of the three positive rapid diagnostic tests performed at the time of HAT diagnosis in September 2017. (TIF)

S2 Fig Video of the single trypanosome detected by the mAECT parasitological test confirming the HAT status of the patient in September 2017. (MP4)
