# Supplementary figures and images for: The complex health seeking pathway of a human African trypanosomiasis patient in Côte d’Ivoire underlines the need of setting up passive surveillance systems
Source: PLoS Negl Trop Dis. 2020 Sep 14;14(9):e0008588. doi: 10.1371/journal.pntd.0008588 (PMC7515183; doi:10.1371/journal.pntd.0008588)

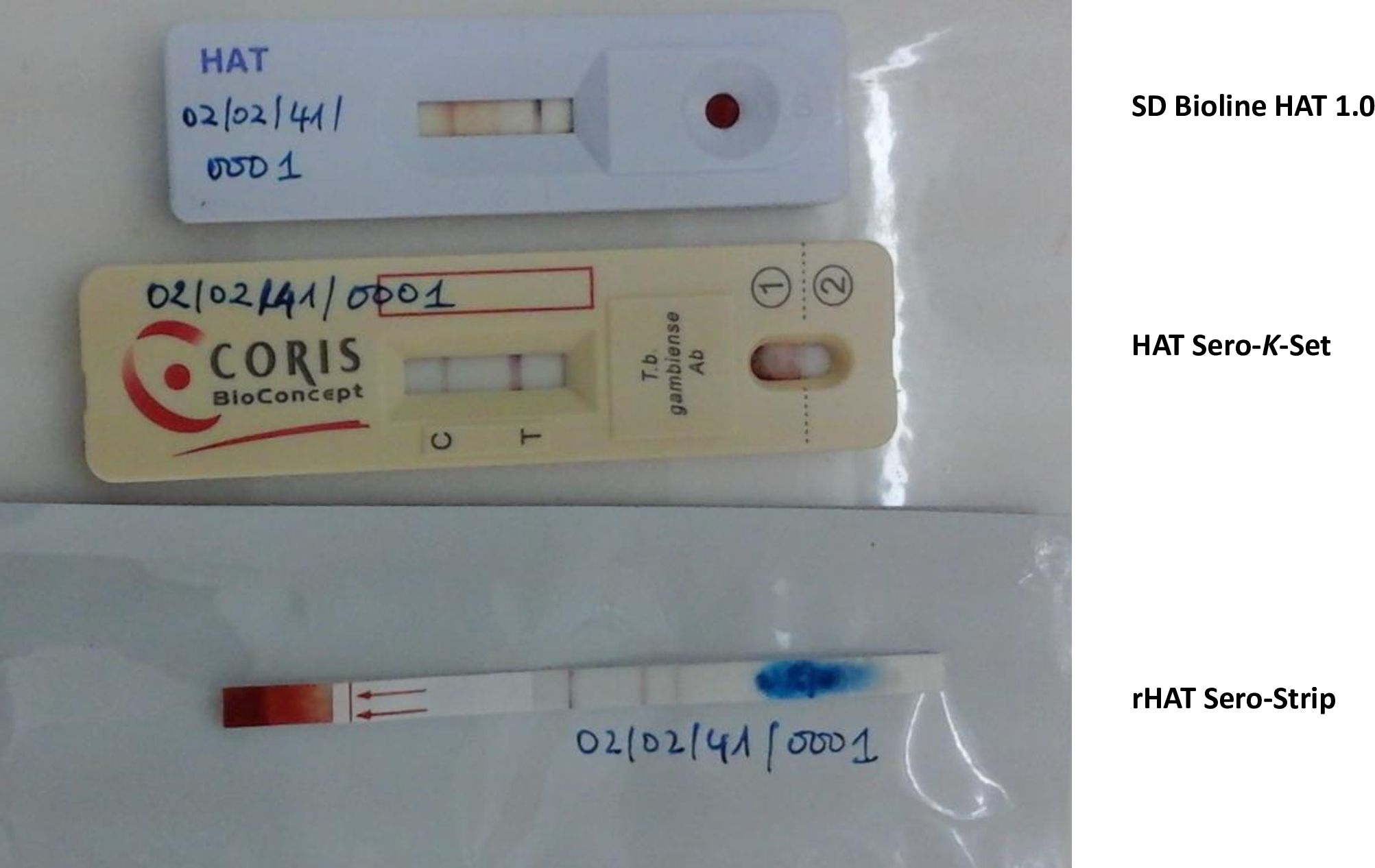

Supplement: S1 Fig — (TIF) [file pntd.0008588.s001.tif]
